# Word Meaning Contributes to Free Recall Performance in Supraspan Verbal List-Learning Tests

**DOI:** 10.3389/fpsyg.2020.02043

**Published:** 2020-08-14

**Authors:** Sandrine Cremona, Gaël Jobard, Laure Zago, Emmanuel Mellet

**Affiliations:** Groupe d’Imagerie Neurofonctionnelle, IMN, UMR 5293, CNRS, Univ. Bordeaux, CEA, Bordeaux, France

**Keywords:** Rey Auditory Verbal Learning Test (RAVLT), semantic memory, self-reported strategies, adult healthy human volunteers, BIL&GIN

## Abstract

Supraspan verbal list-learning tests, such as the Rey Auditory Verbal Learning Test (RAVLT), are classic neuropsychological tests for assessing verbal memory. In this study, we investigated the impact of the meaning of the words to be learned on three memory stages [short-term recall (STR), learning, and delayed recall (DR)] in a cohort of 447 healthy adults. First, we compared scores obtained from the RAVLT (word condition) to those of an alternative version of this test using phonologically similar but meaningless items (pseudoword condition) and observed how each score varied as a function of age and sex. Then, we collected the participants’ self-reported strategies to retain the word and pseudoword lists and examined if these strategies mediated the age and sex effects on memory scores. The word condition resulted in higher memory scores than pseudoword condition at each memory stage and even canceled out, for the learning stage, the detrimental effect of age that was observed for the short-term and DR. When taking sex into account, the word advantage was observed only in women for STR. The self-reported strategies, which were similar for words and pseudowords, were based on the position of the item on the list (word: 53%, pseudoword: 37%) or the meaning of the item (word: 64%, pseudoword: 58%) and were used alone or in combination. The best memory performance was associated with the meaning strategy in the word condition and with the combination of the meaning and position strategies in the pseudoword condition. Finally, we found that the word advantage observed in women for STR was mediated by the use of the meaning strategy. The RAVLT scores were thus highly dependent on word meaning, notably because it allowed efficient semantic knowledge-based strategies. Within the framework of Tulving’s declarative memory model, these results are at odds with the depiction of the RAVLT as a verbal *episodic memory* test as it is increasingly referred to in the literature.

## Introduction

Memory is among the cognitive functions that change the most with aging. With the current aging of the population, the study of memory during normal and pathological aging has become a major focus in the neuroscience field (Park and Festini, 2017). Neuropsychological assessment of learning and memory in aging populations frequently relies on the use of learning tasks based on supraspan word-lists (i.e., list lengths that exceed memory span), such as those from the Consortium to Establish a Registry for Alzheimer’s Disease (CERAD; Morris et al., 1989), the California Verbal Learning Test (CVLT; Delis et al., 1987) or the Rey Auditory Verbal Learning Test (RAVLT; Rey, 1970). These tests include measures of short- and long-term recall and recognition, vulnerability to proactive and retroactive interference and learning ability. Although the CVLT yields more information, particularly regarding the encoding strategies used by the participants (Stricker et al., 2002), the RAVLT has long offered more adequate normative data and alternative test forms (Spreen et al., 1998). As reviewed by Saury and Emanuelson (2017), the extended version of the RAVLT can distinguish among the following four domains of learning and memory: (a) short-term recall (STR), (b) learning, (c) interference, and (d) retrieval [immediate recall after interference, delayed recall (DR), and delayed recognition]. Thus, the RAVLT provides numerous memory scores and derivative indices (Spreen et al., 1998). At the neuropsychological level, RAVLT performance (STR, learning, and DR) can discriminate among normal aging, mild cognitive impairment (MCI) and Alzheimer’s disease (AD; Marra et al., 2000; Estévez-González et al., 2003; Goryawala et al., 2015; Bauer et al., 2018) and is a good independent predictor of dementia (Eckerström et al., 2013; Li et al., 2017). Moreover, RAVLT DR performance can discriminate AD from other dementias, such as the behavioral variant of frontotemporal dementia (Ricci et al., 2012) or dementia with Lewy bodies (Bussè et al., 2017). Therefore, the clinical use of the RAVLT to detect memory impairment is supported by the French social security system (ALQP006 CCAM). Taken together, these elements can explain why the RAVLT is currently highly popular as shown by its inclusion in cognitive batteries used for aging studies [e.g., the Alzheimer’s Disease Neuroimaging Initiative (ADNI, Park et al., 2012) and the Canadian Longitudinal Study on Aging (CLSA, Tuokko et al., 2017)].

The present study aimed to further characterize the role of verbal material, i.e., its meaning, in memory assessment using supraspan verbal list-learning tests such as the RAVLT. To achieve our goal, we adopted a two-pronged approach that involved a healthy adult population of 447 participants under the age of 60 to avoid the pronounced effects of aging on memory.

First, to study the role of the lexico-semantic component of the verbal material, we compared memory scores obtained from the RAVLT to those of an alternative version of this test using phonologically similar but meaningless items, i.e., pseudowords. To the best of our knowledge, this issue has not been explored in this manner since certain preliminary works were carried out by Ebbinghaus on himself and by Tulving on a group of six participants (Tulving, 1985). Pseudowords are orthographically legal and pronounceable letter strings without meaning that should mainly activate orthographic and phonological sources of information but only partially (or not at all) lexical information (Mazoyer et al., 1993; Ziegler et al., 1997). Therefore, we were able to evaluate the lexico-semantic benefit by comparing pseudoword from word memory performance. As a first assumption, we expected that word meaning would enable better word than pseudoword memory performance. Moreover, demographic factors have been shown to have a major impact on RAVLT scores in the following manner: performance declines during healthy aging (McMinn et al., 1988; Geffen et al., 1990; Selnes et al., 1991; Mortensen and Gade, 1993; Van Der Elst et al., 2005; Malloy-Diniz and Parreira, 2007; Messinis et al., 2007; Teruya et al., 2009), and women perform better than men (Bleecker et al., 1988; Geffen et al., 1990; Mortensen and Gade, 1993; Aartsen et al., 2004; Maitland et al., 2004; Malloy-Diniz and Parreira, 2007; Messinis et al., 2007). We therefore examined the variability of the RAVLT and the pseudoword memory scores as a function of age and sex.

Second, we collected the participants’ self-reports of the strategies they used during the task to assess whether the self-reported strategies could lead to a better understanding of the explicit mechanisms by which the learning and memory occur. The use of encoding strategies has been linked to greater recall in word-list learning tests (Unsworth et al., 2019). Compared to the CVLT, in which the words are experimentally grouped into four categories to encourage the use of a semantic clustering strategy (Sunderaraman et al., 2013), the RAVLT words do not have a clear semantic relationship. Some authors have argued that serial order might become a preferred strategy in such cases (Vakil and Blachstein, 1994; Meijs et al., 2013). Here, we investigated the nature of the participants’ declarative memory processes without any preconception by collecting their self-reported strategies. Therefore, our second hypothesis was that RAVLT performance could be influenced by the self-reported strategies that the participants used. We also investigated whether these strategies could mediate the well-known effect of age and sex on RAVLT performance.

## Materials and Methods

### Participants

The present study included 447 healthy volunteers recruited during the same period whose native language tongue was French and who had no past history of neuropsychiatric disorders. The data were derived from the BIL&GIN database (Mazoyer et al., 2016), and we excluded four participants with unavailable data and two participants with suspected dyslexia. The experimental protocol was approved by the local ethics committee (Comité de Protection des Personnes Nord-Ouest). All participants provided written informed consent and received compensation for their participation. The study sample comprised 228 women and 219 men with a mean age of 26.6 years (range from 18 to 58 years) and was balanced for handedness. The mean level of education was 15.3 years, which corresponds to 3 years of university education.

### Cognitive Tests

Data were extracted from the standardized battery of 10 cognitive tests in the BIL&GIN, a database acquired by the team and previously described in detail (Mazoyer et al., 2016). Briefly, the participants completed 10 cognitive tests distributed over two sessions separated by an approximately 3-h MRI acquisition period. We used six verbal tests that were ordered within each cognitive session as follows:

– Session 1: the RAVLT, vocabulary scope, a rhyme judgment task and the listening span test.
– Session 2: the pseudoword memory test and the reading span test.

#### Assessment of the Memory of Words (RAVLT) and Pseudowords

Since the RAVLT has severe ceiling effects in healthy young adults (i.e., those 18–39 years of age, Uttl, 2005), we attempted to limit the ceiling effect by increasing the number of words to be learned. In total, 18 words were used (livre, fleur, train, tapis, prairie, harpe, sel, jardin, doigt, tambour, pomme, cheminée, rivière, bouton, clé, chien, verre, and hochet, Rey, 1970; Lezak, 1983) instead of the 15 words used in the common version. The verbal memory test consisted of listening to a list of concrete “unrelated” words (one word per second read aloud by the experimenter in the same order in each of the five consecutive trials). Immediately after each trial, the participants were instructed to freely recall as many items as possible. After a 20-min delay (during which a nonverbal task was performed), the participants were asked to recall this list again. A similar procedure was applied using 15 pseudowords created with the WordGen software (Duyck et al., 2004) and matched to the list of words by the number of letters, phonemes, syllables and bigram frequencies (guice, anire, ficot, meple, flaxion, jaron, asue, ecrot, diare, doussant, boidir, sato, fince, veigne, and gouage).

As the measures were collected in a healthy adult population, we expected a relatively high level of DR (Van Der Elst et al., 2005). Additionally, since recognition requires fewer processing resources than recall (Craik and McDowd, 1987), we did not include a recognition task and focused instead on three scores (Ivnik et al., 1990) corresponding to the following three stages of memory:

1. STR: recall score in trial 1;
2. Learning over trials (LOT): the sum of the items recalled over trials 2 & 3 minus 2 times the STR (Ivnik et al., 1990; Teruya et al., 2009); and
3. DR: the long-term percent retention was calculated as the delayed trial six score divided by the maximum score achieved during one of the first five learning trials × 100 (Moradi et al., 2017).

The detailed method for determining the most suitable scores for evaluating each memory stage is described in the Supplementary Material. To avoid any ceiling effect, Uttl advised that the mean of each score be distanced from the maximum score by more than one SD (Uttl, 2005). Thus, we checked that all mean memory scores met this criterion.

#### Assessment of Confounding Factors

##### Vocabulary scope

Greater vocabulary and verbal IQ have been associated with better RAVLT performance (Bolla-Wilson and Bleecker, 1986; Mortensen and Gade, 1993). A synonym-finding test served to estimate the extent of vocabulary (Binois and Pichot, 1956). Across 44 trials (max score), the participants had to determine which of the six written words was synonymous with a target word presented at the top of the screen.

##### Rhyme judgment task (rhyming)

Phonological abilities could impact pseudoword processing. To estimate phonological abilities, a rhyme judgment task (adapted from Shaywitz et al., 1995) was completed using 80 pairs of pseudowords. The pairs of pseudowords were presented and remained on the screen until the participant indicated by pressing a key whether or not they rhymed or for a maximum of 4 s. The pseudowords were composed of 1–3 syllables (3–9 letters); 30 pairs rhymed, while 50 did not, leading to a maximum possible score of 80. To prevent the use of strategies based simply on visually matching between the ends of the pseudowords, 45 items were constructed so that the visual information conflicted with the expected responses (i.e., by using pseudowords with similar spellings but different sounds or pseudowords with different spellings but same sounds).

##### Working memory capacity

Working memory capacity (WMC) has been shown to mediate the rate of age-related decline in verbal memory (Constantinidou et al., 2014). Here, WMC was evaluated with two complex working memory span tasks: the French adaptation of the reading span test and its auditory counterpart, the listening span test (Daneman and Carpenter, 1980; Desmette et al., 1995). During the reading span test, the participant had to read aloud blocks of sentences presented on a computer screen. The number of sentences per block started at 2 and increased by 1 sentence every 3 blocks until 6 sentences were presented. At the end of each block, the participant had to remember the last word of each sentence while avoiding starting with the last sentence. The same pattern was used for the listening span test but with 2 exceptions, as follows: each sentence was read by the examiner, and the participants had to determine whether the sentence was in the present tense. According to the Daneman and Carpenter method, truncated spans were scored for each test by starting with the highest level (2–6) at which the participant recalled the majority of the blocks (2 out of 3) and adding half a point for recalling 1 out of 3 blocks at the subsequent level. The truncated spans for reading and listening were averaged to obtain the WMC score.

Vocabulary scope, rhyming task and reading span test were conducted with E-Prime (Version 2, Pittsburgh, PA: Psychology Software Tools.).

### Collection and Categorization of Self-Reported Strategies

Self-reported strategies were collected after the word and pseudoword DR tasks by a psychologist trained in interviewing. The self-reported strategies corresponded to all three memory stages (STR, LOT, and DR). The participants were asked to explain how they retained the lists, and their answers were recorded as field notes. An open question was used to avoid influencing the nature of the answers. At this stage, data related to words from three participants were missing, as were data related to pseudowords from five participants.

Based on the psychologist’s field notes, two experimenters compiled an exhaustive list of the strategies used for the words and pseudowords and standardized the wording of the strategies on the basis of consensus. This standardization was facilitated by the fact that the psychologist’s field notes were already partially standardized. A set of categories that covered all strategies and allowed word and pseudoword comparisons was obtained according to the following rules (Table 1):

**TABLE 1:** Frequency of use and categorization criteria for the set of standardized self-reported word and pseudoword strategies.

**Words**	**N**	**Involves an auditory representation**	**Involves rethinking the order of the list**	**Implies knowledge about the meaning of the items**	**Refers to the participants’ time-space**	**Categorization of the words and pseudowords self-reported strategies**	**Refers to the participants’time-space**	**Implies a phoneme-to-semantic system conversion**	**Involves rethinking the order of the list**	**Involves an auditory representation**	**N**	**Pseudowords**
No/few strategy	10	**X**				**Listening**				**X**	10	No/few strategy
(only) Listening	79	**X**								**X**	70	(only) Listening
Rehearsal	31	**X**								**X**	26	Rehearsal
Sound/sonorities	31	**X**								**X**	56	Sound/sonorities
Rhymes	9	**X**								**X**	2	Rhymes

Ordered rehearsal	5	**X**	**X**			**Position**			**X**	**X**	6	Ordered rehearsal
Bundled	15	**X**	**X**						**X**	**X**	9	Bundled
Ordered, by rote	146	**X**	**X**						**X**	**X**	107	Ordered, by rote
First/beginning	103	**X**	**X**						**X**	**X**	50	First/beginning
Last/end	75	**X**	**X**						**X**	**X**	43	Last/end

Taxonomic categorization	87	**X**		**X**		**Meaning**		**X**		**X**	251	Association with real/known words
Thematic relation	130	**X**		**X**								
Image/visualization	67	**X**		**X**								
Story	9	**X**		**X**								

Recollection	11	**X**			**X**	**Autonoetic**	**X**			**X**	1	Recollection
Test room item	3	**X**			**X**		**X**			**X**	0	Test room item

1 Since an auditory representation of the items was directly induced by the auditory presentation of the lists, Listening was the default strategy.
2 If a strategy involved rethinking the order of the list, it was considered a Position strategy.
3 If a strategy implied knowledge about the meaning of the items (words) or a phoneme-to-semantic system conversion (pseudowords), it was considered a Meaning strategy.
4 If a strategy referred to the participant’s time-space, it was considered an Autonoetic strategy.

Finally, the participants were categorized into four types of strategy use as follows:

1 Those who only used Listening strategies (words *n* = 67; pseudowords *n* = 110);
2 Those who only used Position strategies (words *n* = 93; pseudowords *n* = 80);
3 Those who only used Meaning strategies (words *n* = 142; pseudowords *n* = 168);
4 Those who used both Position and Meaning strategies, which was labelled Dual (words *n* = 142; pseudowords *n* = 84).

The Listening strategy that was directly induced by the auditory presentation of the items was regarded as a *minimal* strategy compared to the other 3, which were considered more *advanced*. Moreover, since autonoetic strategies were used by only 3% of the participants and were never used alone, except by one participant, the Autonoetic category was not separately addressed as a strategy type in further analysis.

### Data Analysis

The statistical analyses were conducted in R Version 3.5.2.^1^ The figures were created with the following packages: *ggplot2* (Wickham, 2016) and *interactions* (Long, 2019). The alpha level of 0.05 or the 95% confidence interval (CI) was used to determine the significance of the hypothesis tests.

#### Group Comparisons of the Word and Pseudoword Memory Scores

Each memory score (STR, LOT, and DR) was averaged across participants, and the mean scores for the words and pseudowords were compared with a paired sample *t*-test Bonferroni corrected for the multiplicity of tests (*p* < 0.016). Each individual memory score was further standardized, thereby allowing a direct comparison in the subsequent analyses.

#### Modeling Memory Performance by Age and Sex

To estimate the effects of age and sex on each memory stage of words and pseudowords, we implemented a linear mixed model (*lmerTest::lmer*, Kuznetsova et al., 2017). A 4-way interaction among age ^∗^ sex ^∗^ type of item (2 levels: words and pseudowords) ^∗^ memory stage (3 levels: STR, LOT, and DR) was introduced in the model as a fixed effect. We further included the following variables as confounders: vocabulary scope, rhyming, WMC, level of education (Selnes et al., 1991; Van Der Elst et al., 2005; Malloy-Diniz and Parreira, 2007; Messinis et al., 2007; Teruya et al., 2009; Magalhães and Hamdan, 2010), and handedness (Mellet et al., 2013). The random effects of type of item and memory stage were fitted on the intercept at the participant level. A stepwise backward strategy for model selection based on the Akaike information criterion (AIC) was applied to the linear mixed model described above (*lmerTest::step*, Kuznetsova et al., 2017) to determine the best-fitted model. The ANOVA components were calculated based on the final model with the Kenward-Roger approximation (Halekoh and Højsgaard, 2014) to correct for the underestimation of variance due to sampling fluctuations. The residuals were visually inspected to assess normality and homoscedasticity. The proportion of variance explained by the fixed effects and their interactions was estimated with the marginal *R*^2^, as described in Nakagawa et al. (2017). All pairwise *post hoc* comparisons were corrected for multiple comparisons using an FDR-controlling procedure (*emmeans*, Lenth, 2020).

#### Analyses of Self-Reported Strategies

The distributions of the self-reported strategies, which were separately reported for the pseudowords and words, were compared with the test of marginal homogeneity, an extension of McNemar’s test for dependent samples for multilevel variables. For each strategy, pairwise *post hoc* comparisons between the word and pseudoword proportions were performed with McNemar’s test with Bonferroni correction for multiple tests.

Then, we investigated whether the studied variables, i.e., age and sex, impacted the occurrence of each self-reported strategy. Therefore, two multinomial log-linear models were assessed (one model for words and one model for pseudowords) with the four self-reported strategies as the multiclass outcome and the two variables of interest as the predictors (*nnet::multinom* function, Venables and Ripley, 2002).

#### Modeling Memory Performance by Self-Reported Strategies

To estimate the effects of the self-reported strategies on word and pseudoword memory performance at each memory stage, we implemented a linear mixed model. A 3-way interaction of strategy (4 levels: Listening, Position, Meaning, and Dual) ^∗^ type of item (2 levels: words and pseudowords) ^∗^ memory stage (3 levels: STR, LOT and DR) was introduced in the model as a fixed effect. The random effects of type of item and memory stage were fitted on the intercept at the subject level. A stepwise backward strategy for model selection using the AIC was applied to the linear mixed model described above to determine the best-fitted model. The ANOVA components were calculated using the Kenward–Roger approximation. The residuals were visually inspected to assess normality and homoscedasticity. The proportion of variance explained by the fixed effects and their interactions was estimated with the marginal *R*^2^. All pairwise *post hoc* comparisons were corrected for multiple comparisons using an FDR-controlling procedure.

#### Causal Mediation Analysis (CMA)

Causal mediation analysis was conducted to investigate the contribution of the self-reported strategies to the relationships between age or sex and memory scores. Causal mediation analysis decomposes the total effect of a predictor into direct (i.e., the effect of the predictor on the dependent variable adjusted for the predictor–mediator relationship) and indirect (i.e., the mediator effect) effects. The MBESS library was implemented (*MBESS::mediation*, Kelley, 2019) with a bias-corrected bootstrapped 95% CI with 10,000 samples. The effect size was estimated by kappa squared (κ^2^), which corresponds to the proportion of the maximum possible indirect effect that could have occurred (Preacher and Kelley, 2011). Since the first method of calculation was criticized (Wen and Fan, 2015), here, κ^2^ was calculated according to Talloen’s proposition (Talloen W. Effect size measures for mediation models: a critical evaluation of κ2. Unpublished results, 2015) and implemented in R with the *MaxIE* function provided by the author.

## Results

### Characterization of Words Memory Scores and Comparison With Pseudoword Memory Scores

First, we characterized and compared the memory scores of the words with those of the pseudowords. The first result was the number of recalled words that was significantly higher than the number of pseudowords at each memory stage, corresponding to a generalized semantic benefit (Table 2).

**TABLE 2:** Descriptive data of the studied sample and memory measures.

**Studied sample (*N* = 447)**	**Mean (SD)/% (*n*)**	**[Range]**
Age (years)	26.64 (*7.60*)	[18.09–57.22]
Sex	W: 51 % (*228*)	
	M: 49 % (*219*)	
**Confounding factors**		
Handedness	Left: 45.41 % (*203*)	
	Right: 54.59 % (*244*)	
Education (years)	15.27 (*2.50*)	[8–20]
Vocabulary scope (max. 44)	27.47 (*4.25*)	[15–36]
Rhyming (max. 80)	67.02 (*6.08*)	[44–77]
WMC (max. 6)	4.17 (*0.99*)	[2–6]
**Verbal list test (RAVLT)**		
STR (trial 1, max. 18)	7.99 (*2.00*)*	[2–16]
LOT (trials 2 + 3)−(trial 1 × 2)	9.72 (*3.21*)*	[1–18]
DR (% of max. trial)	90.49 (*10.80*)*	[50–121.43]
**Pseudoword list test**		
STR (trial 1, max. 15)	2.91 (*1.40*)	[0–9]
LOT (trials 2 + 3)−(trial 1 × 2)	6.97 (*3.78*)	[-2 to 17]
DR (% of max. trial)	62.30 (*24.73*)	[0 - 118.18]

Continuous variables are summarized as the mean ± SD, and categorical variables are summarized as % (*n*); WMC, working memory capacity; STR, short-term recall; LOT, learning over trials; DR, delayed recall; *significant differences between word and pseudoword scores, as assessed by a paired samples Bonferroni *t*-test corrected for the multiplicity of tests (*p* < 0.016). *nota bene*: The same significant differences were observed when STR and LOT were expressed as the percentage of list length.

### Effects of Age and Sex on Memory Performance

After adjusting for education, vocabulary scope, rhyming, WMC, and handedness, the effects of age and sex on the three memory stages of the words and pseudowords were modeled with a linear mixed model. The final model, which was obtained by a stepwise backward strategy, explained 27% of the variance, and 13.3% of the variance was due to fixed-effect factors.

#### Age Effect on Pseudoword and Word Memory According to the Memory Stage

**FIGURE 1:**
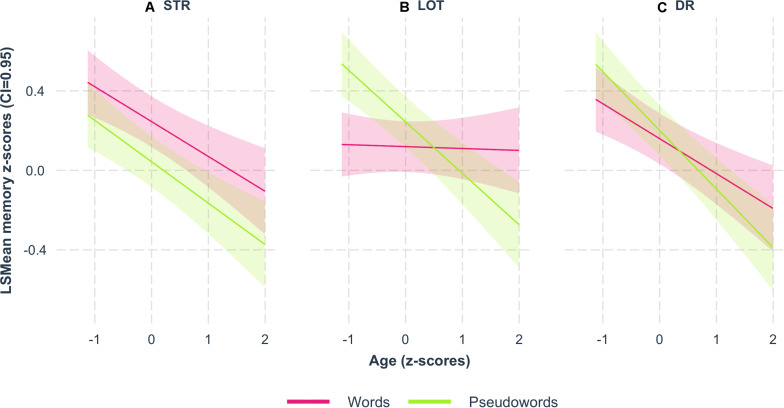
Memory scores as a function of age. Age interaction with the type of item (pink: words; green: pseudowords) and 3 memory stages: **(A)** short-term recall – STR, **(B)** learning over trials – LOT, and **(C)** delayed recall – DR. All memory scores (LSmean of z-scores adjusted for sex, education, vocabulary scope, rhyming, WMC, and handedness) significantly decreased with age, except for the LOT of the words which remained stable.

A 3-way interaction among age, type of item and memory stage was observed (F_(2,1332)_ = 3.61; *p* = 0.03; Figure 1 and Table 3). A general decline in performance with age was visible in the 3 pseudoword memory stages but was limited to STR and DR for word memory. Thus, with increasing age, word meaning improved word memory scores relative to pseudoword memory scores for LOT (ß = 0.249, *t*_(1, 1332)_ = 4.44, *p* < 0.0001) and, to a lesser extent, for DR (ß = 0.119, *t*_(1, 1332)_ = 2.07, *p* = 0.04).

**TABLE 3:** Memory performance as a function of age for words and pseudowords at each memory stage after adjusting for sex, education, vocabulary scope, rhyming, WMC, and handedness.

	**STR: short-term recall**				**LOT: learning over trials**				**DR: delayed recall**			
**(A) Age (+1SD)**	***ß***	***SE***	***t*_(2508)_**	***p***	***ß***	***SE***	***t*_(2508)_**	***p***	***ß***	***SE***	***t*_(2508)_**	***p***
Words	–0.175	0.04	–3.92	0.0001	–0.010	0.04	–0.21	0.831	–0.176	0.04	–3.93	0.0001
Pseudowords	–0.208	0.04	–4.65	<0.0001	–0.259	0.04	–5.78	<0.0001	–0.295	0.04	–6.58	<0.0001

**(B) Words > Pseudowords**	***ß***	***SE***	***t*_(1332)_**	***p***	***ß***	***SE***	***t*_(1332)_**	***p***	***ß***	***SE***	***t*_(1332)_**	***p***

	0.033	0.05	0.57	0.567	0.249	0.05	4.44	<0.0001	0.119	0.05	2.07	0.038

*(A) FDR-corrected *post hoc* estimation of the word and pseudoword slopes associated to 1 SD of age. (B) The FDR-corrected *post hoc* pairwise comparisons of the word and pseudoword slopes revealed a significant difference for LOT and DR.*

#### Sex Effect on Pseudoword and Word Memory According to the Memory Stage

**FIGURE 2:**
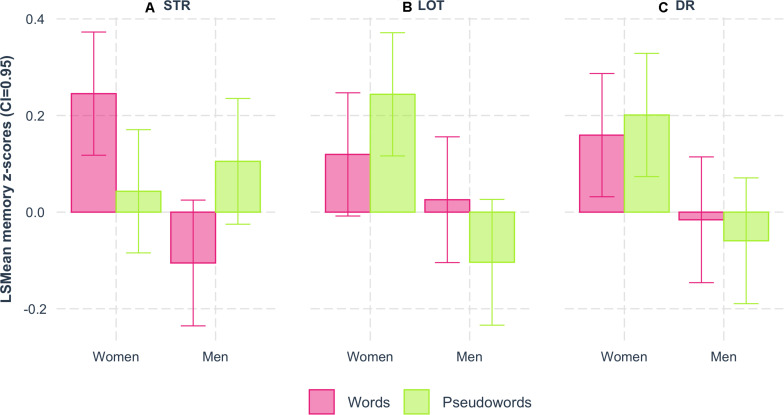
Memory scores as a function of sex. Sex interaction with the type of item (pink: words; green: pseudowords) and memory stages: **(A)** short-term recall – STR, **(B)** learning over trials – LOT, and **(C)** delayed recall – DR. Memory scores (LSmean of z-scores adjusted for age, education, vocabulary scope, rhyming, WMC, and handedness) were significantly higher for words than for pseudowords among women at the STR.

A 3-way interaction among sex, type of item, and memory stage was observed (*F*_(2,1332)_ = 9.12; *p* < 0.0001; Figure 2 and Table 4). The word memory scores were significantly higher than the pseudoword memory scores exclusively among women for STR (FDR-corrected *post hoc* test: words: *t*_(1,1332)_ = 2.51, *p* = 0.012). In contrast, the STR of pseudowords was higher than the STR of words among men (FDR-corrected *post hoc* test: words: *t*_(1, 1332)_ = −2.57, *p* = 0.010). From the perspective of sex contrasts, women performed significantly better than men on word STR and DR (FDR-corrected *post hoc* tests: STR: *t*_(1, 2541)_ = 3.95, *p* < 0.0001; DR: *t*_(1, 2541)_ = 1.97, *p* = 0.049) and on pseudoword LOT and DR (FDR-corrected *post hoc* tests: LOT: *t*_(1,2541)_ = 3.92, *p* < 0.0001; DR: *t*_(1, 2541)_ = 2.93, *p* = 0.003).

**TABLE 4:** Memory performance as a function of sex and its interaction with the type of item and memory stage after adjusting for age, education, vocabulary scope, rhyming, WMC, and handedness.

**Sex**	**STR: short-term recall**				**LOT: learning over trials**				**DR: delayed recall**			
**(A) Words > Pseudowords**	***ß***	**SE**	***t*_(1332)_**	***p***	***ß***	**SE**	***t*_(1332)_**	***p***	***ß***	**SE**	***t*_(1332)_**	***p***
Women	0.202	0.08	2.51	0.012	–0.124	0.08	–1.55	0.121	–0.042	0.08	–0.52	0.603
Men	–0.210	0.08	–2.57	0.010	0.129	0.08	1.58	0.114	0.043	0.08	0.53	0.596

**(B) Women > Men**	***ß***	**SE**	***t*_(2541)_**	***p***	***ß***	**SE**	***t*_(2541)_**	***p***	***ß***	**SE**	***t*_(2541)_**	***p***

Words	0.35	0.06	3.95	<0.0001	0.094	0.06	1.06	0.291	0.175	0.06	1.97	0.049
Pseudowords	–0.062	0.06	–0.69	0.485	0.348	0.06	3.92	<0.0001	0.260	0.06	2.93	0.003

*(A) FDR-corrected pairwise comparisons of word and pseudoword memory performance among women and men at each memory stage. (B) FDR-corrected pairwise comparisons of women’s and men’s memory performance for words and pseudowords at each memory stage.*

### Characterization of the Self-Reported Strategies

The distribution of the four self-reported strategies for the memory of words (*N* = 444) and pseudowords (*N* = 442) is shown in Table 5.

**TABLE 5:** Self-reported strategy distribution according to the type of item.

**Strategy type**	**Pseudowords (%)**	**Words (%)**	**McNemar’s χ^2^**	***p***
Listening	24.3	14.1	24.40	<0.0001
Position	18.0	21.1	2.23	0.5424
Meaning	38.4	32.3	5.17	0.0919
Dual (Position + Meaning)	19.3	32.5	27.13	<0.0001

*The differences in the proportions of strategy use between pseudowords and words were tested with McNemar’s Bonferroni test corrected for four tests.*

Strikingly, the strategies used to retain the list of pseudowords were primarily Meaning based, as follows: 38.4% of the participants used the Meaning strategy, and 19.3% of the participants used this strategy in association with the Position strategy. The Position strategy alone was used by 18% of the participants. For the RAVLT, the dominant strategy was Meaning, as 32.3% of the participants used this strategy alone, 32.5% of the participants used this strategy in association with the Position strategy, and 21.1% of the participants exclusively used the Position strategy. The distribution of the pseudoword strategies significantly differed from the distribution of word strategies (McNemar’s χ^2^_(6)_ = 24.40, *p* < 0.0001). Compared to the words, the pseudowords elicited a greater use of the Listening strategy (McNemar’s χ^2^_(1)_ = 27.13, *p* < 0.0001) and less use of the Dual strategy (McNemar’s χ^2^_(1)_ = 55.65, *p* < 0.0001).

### Effects of the Self-Reported Strategies on Memory Performance

The effects of the self-reported strategies on the 3 stages of word and pseudoword memory were modeled in a linear mixed model. The final model, which was obtained by a stepwise backward strategy, explained 28% of the variance, and 5.6% of the variance was attributable to fixed-effect factors.

**FIGURE 3:**
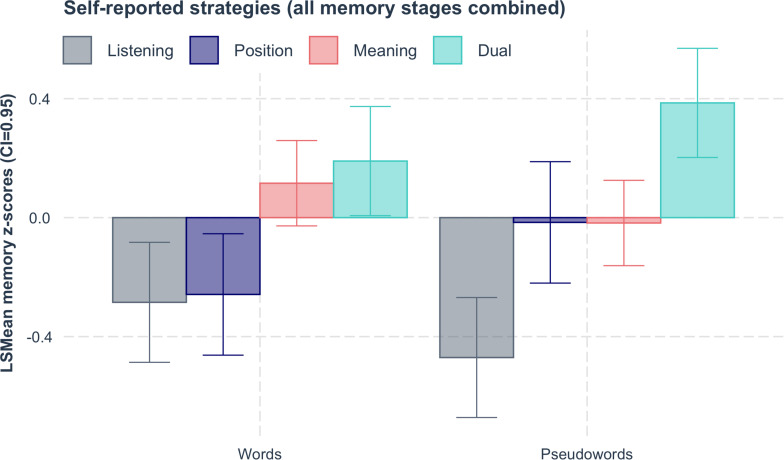
Effect of the self-reported strategies on word and pseudoword memory performance.

The interaction between the self-reported strategies and the type of item had a major significant effect (F_(3,662)_ = 4.87; *p* = 0.002), which is clearly visible in Figure 3 and Table 6. Indeed, the Meaning and Dual strategies both improved word memory performance compared to the Listening strategy (respective FDR-corrected *post hoc* tests: *t*_(1,401)_ = 3.17, *p* = 0.003 and *t*_(1, 401)_ = 3.42, *p* = 0.003) and the Position strategy (respective FDR-corrected *post hoc* tests: *t*_(1, 401)_ = 2.94, *p* = 0.005 and *t*_(1, 401)_ = 3.21, *p* = 0.003). Concerning the pseudowords, all 3 advanced strategies improved memory performance relative to the Listening strategy (FDR-corrected *post hoc* tests: Position: *t*_(1, 401)_ = 3.11, *p* = 0.003, Meaning: *t*_(1, 401)_ = 3.58, *p* = 0.001, Dual: *t*_(1, 401)_ = 6.16, *p* < 0.0001). Moreover, regarding the pseudowords, use of the Dual strategy significantly increased the memory score compared to the Position strategy (FDR-corrected *post hoc* tests: *t*_(1, 401)_ = 2.87, *p* = 0.005) and the Meaning strategy (FDR-corrected *post hoc* tests: *t*_(1, 401)_ = 3.40, *p* = 0.001), suggesting that a potentiating effect occurs when the Position and Meaning strategies are combined.

**TABLE 6:** Memory performance as a function of self-reported strategy and its interaction with the type of item.

**Strategies**	**Position**				**Meaning**				**Dual**			
	***ß***	***SE***	***t*_(401)_**	***p***	***ß***	***SE***	***t*_(401)_**	***p***	***ß***	***SE***	***t*_(401)_**	***p***
**(A) Listening<**												
Words	0.026	0.15	0.18	0.856	0.400	0.13	3.17	0.003	0.475	0.14	3.42	0.003
Pseudowords	0.454	0.15	3.11	0.003	0.452	0.13	3.58	0.001	0.856	0.14	6.16	<0.0001
**(B) Position<**												
Words					0.374	0.13	2.94	0.005	0.448	0.14	3.21	0.003
Pseudowords					–0.002	0.13	–0.02	0.987	0.402	0.14	2.87	0.005
**(C) Meaning<**												
Words									0.074	0.12	0.63	0.636
Pseudowords									0.404	0.12	3.40	0.001

*FDR-corrected pairwise comparisons of memory performance for each strategy for words and pseudowords: (A) Listening as ref., (B) Position as ref., (C) Meaning as ref.*

### Mediation of the Age and Sex Effect on Memory Performance by Self-Reported Strategies

First, we assessed the impact of age and sex on the likelihood of using the 4 self-reported strategies compared to each other. The main outcomes are summarized in Table 7. Relative to use of the Listening strategy, the likelihood of using any of the three advanced strategies to retain both the words and pseudowords decreased with age. Moreover, comparisons to the Position strategy revealed an influence of sex. Regarding words, being a woman increased the likelihood of using the Meaning and the Dual strategies. Regarding pseudowords, being a woman increased the likelihood of using the Meaning strategy.

**TABLE 7:** Adjusted odds ratio (aOR) and 95% confidence interval (CI) derived from the multinomial logistic model comparing each strategy to the Listening and Position strategies.

**Words**				**Pseudowords**			
	**LISTENING as ref.**	**aOR**	**95% CI**		**LISTENING as ref.**	**aOR**	**95% CI**
**Age**	**Position**	**0.95**	**0.91–0.98**	**Age**	**Position**	**0.95**	**0.92–0.99**
	**Meaning**	**0.96**	**0.92–0.99**		**Meaning**	**0.95**	**0.92–0.98**
	**Dual**	**0.93**	**0.90–0.97**		**Dual**	**0.92**	**0.88–0.97**
**Women**	Position	0.83	0.43–1.62	**Women**	Position	0.83	0.46–1.52
	Meaning	1.52	0.82–2.81		Meaning	1.46	0.88–2.42
	Dual	1.7	0.91–3.18		Dual	1.31	0.72–2.38

	**POSITION as ref.**	**aOR**	**95% CI**		**POSITION as ref.**	**aOR**	**95% CI**

**Age**	**Listening**	**1.06**	**1.01–1.10**	**Age**	**Listening**	**1.05**	**1.01–1.09**
	Meaning	1.01	0.97–1.05		Meaning	0.99	0.96–1.04
	Dual	0.98	0.95–1.03		Dual	0.96	0.91–1.02
**Women**	Listening	1.2	0.62–2.34	**Women**	Listening	1.2	0.66–2.18
	**Meaning**	**1.83**	**1.06–3.13**		**Meaning**	**1.74**	**1.01–3.00**
	**Dual**	**2.05**	**1.19–3.52**		Dual	1.56	0.84–2.92

*When the CI excludes the value 1 (in bold), the null hypothesis is rejected, indicating a significantly lower (<1) or higher (>1) likelihood of occurrence compared to the reference level.*

Then, CMA was conducted to assess whether the self-reported strategies mediated the age and sex effects on memory performance (Table 8). Regarding words, the following findings were observed: (1) the sex effect on STR (higher scores among women) was mediated by women’s greater use of the Meaning strategy (used alone or in combination with the Position strategy) and (2) the age effect on STR and DR (decreasing scores with age) was mediated by the reduced use of an advanced strategy (i.e., all strategies but Listening) in older participants. Similarly, the decrease of all pseudoword memory scores with age was mediated by the reduced use of an advanced strategy (i.e., all strategies but Listening). Notably, the effect sizes, as measured by κ^2^, varied between 2.4 and 8.1% of the maximum possible indirect effect and can be considered “small.”

**TABLE 8:** Summary of the significant CMA (causal mediation analyses) of the standardized memory scores at each memory stage; STR, short-term recall; LOT, learning over trials; DR, delayed recall; maxIE, maximum possible indirect effect.

	**Effect**	**ß**	**95% CI**	**maxIE**	**κ^2^**
**Words**					
**Women** → Meaning strategy + Dual strategy → **STR**	Indirect	0.0603	0.0189 to 0.1157	0.74	0.0815
	Direct	0.2599	0.0785 to 0.4412		
**Age** → Advanced strategies → **STR**	Indirect	–0.0296	−0.0644 to −0.0098	–0.8000	0.037
	Direct	–0.192	−0.2845 to −0.0995		
**Age** → Advanced strategies → **DR**	Indirect	–0.0191	−0.0426 to −0.0013	–0.8000	0.0239
	Direct	–0.1993	−0.2924 to −0.1062		
**Pseudowords**					
**Age** → Advanced strategies → **STR**	Indirect	–0.0365	−0.0685 to −0.0154	–0.7821	0.0467
	Direct	–0.2106	−0.3029 to −0.1184		
**Age** → Advanced strategies → **LOT**	Indirect	–0.0455	−0.0817 to −0.0212	–0.7400	0.0615
	Direct	–0.2596	−0.3495 to −0.1696		
**Age** → Advanced strategies → **DR**	Indirect	–0.0472	−0.0849 to −0.0227	–0.7100	0.0665
	Direct	–0.2894	−0.3781 to −0.2006		

## Discussion

As might be expected, compared to learning pseudowords, learning words (known meaningful items) resulted in improved performance at each memory stage, from STR to DR, via LOT (Table 2). Since in supraspan lists, more items are *a priori* more difficult to memorize, the longest length of the list cannot explain the better memory scores for words. This word advantage could arise from a detrimental effect of pseudowords’ novelty, which would have an especially strong impact on STR. However, as the word advantage decreased in the following trials, as shown by the proximity between word and pseudoword LOT, the pseudowords became more familiar with repetition. The word advantage observed in the latest stage of memorization thus indicates that words benefit from an effect other than mere familiarity, one that is probably related to the meaning they carry.

Our observation of the age effects on word memory performance (Table 3 and Figure 1) reproduced numerous previous findings showing that age (16–86 years) was associated with a decrease in both the STR and DR of words but did not have a visible effect on LOT (Geffen et al., 1990; Mitrushina et al., 1991; Poreh, 2005; Van Der Elst et al., 2005). In addition, our results showed that age systematically led to a decrease in pseudoword memory performance for each memory stage. The dissimilar effects of age on word and pseudoword performance were particularly important for LOT suggesting that the word-related gain lasts longer, specifically during the learning process.

We also examined the sex effect since the literature has regularly reported for many years that women perform better than men on STR and DR (Bolla-Wilson and Bleecker, 1986; Geffen et al., 1990; Aartsen et al., 2004; Van Der Elst et al., 2005; Gale et al., 2007; Messinis et al., 2007; Badcock et al., 2011). From the perspective of this sex contrast (Table 4 and Figure 2), our results reproduced these previous findings: women performed better than men on the STR and DR of words, but there was no visible sex difference in word LOT performance (Teruya et al., 2009). Regarding the difference in performance for words and pseudowords, the gain provided by the words emerged only among the women on the STR (Figure 2). This result demonstrates that the word gain can appear during early memory stages, which supplements recent studies highlighting the nature and importance of short-term semantic memory (Campoy et al., 2015; Aizpurua and Koutstaal, 2018).

To better understand the explicit mnemonics involved in word and pseudoword memory, we determined the strategies the participants used to retain the lists of words and pseudowords. We found that words and pseudowords elicited the same types of strategies (Table 1). Two main strategies were reported, namely, a strategy based on associations of meanings (lexical/semantic binding) of the items and a strategy based on the temporal contiguity (position on the list) of the items. These two strategies, called Meaning and Position in the present study, correspond to memory strategies previously described in free recall studies as *subjective* and *serial clustering* (Meijs et al., 2013) or *semantic proximity effects* and *temporal contiguity* (Sederberg et al., 2010). These two strategies were contrasted with a third strategy, namely, the Listening strategy, which has also been previously described in free recall tasks as a *rehearsal* mnemonic technique (Herrmann, 1987). These two strategies were also used in combination, an approach that we called the Dual strategy. Thus, from the complexity perspective, the four strategies could be ordered as follows: the Listening strategy was the simplest, a minimal strategy induced by the item presentation; the Position strategy was equivalent to the Meaning strategy; and the Dual strategy was the most complex strategy, combining two strategies.

Regarding the distribution of the four strategies according to type of item (Table 5), word memory relied on the use of more complex strategies than pseudoword memory, which led to a reduced use of the Listening strategy and an increased use of the Dual strategy. The Meaning strategy alone or in combination was the dominant strategy used for word memory (64% of the respondents) and, quite surprisingly, pseudoword memory (57% of the respondents). The main difference between the words and pseudowords concerned the nature of the Meaning strategy, which consisted of thematic and taxonomic associations for the words and lexical associations for the pseudowords. This result suggests that auditory supraspan list-learning tests encourage memory based on associations with familiar and meaningful knowledge in a hierarchical manner (depending on the cues the item provides), from phonemes to lexicon for pseudowords and from lexicon to semantic binding for words. This interpretation is consistent with a previous finding that participants performed equally well on the RAVLT and CVLT when the semantic categorization of words was experimentally forced in the CVLT (Crossen and Wiens, 1994). We cannot excluded the possibility that the fixed order of list presentation (words followed by pseudowords ∼3 hours later) may have favored the use of a Meaning strategy for pseudowords and constitutes a potential limitation to this study. Nevertheless, the subtypes of the Meaning strategy were not strictly the same for the words and pseudowords (Table 1). Thus, it appears that exposure to known phonological entities, regardless of their meaning, encouraged the use of lexico-semantic associations.

Subsequently, we found that the effectiveness of the self-reported strategies varied between word and pseudoword memory tasks (Table 6 and Figure 3). The Listening strategy led to the lowest word and pseudoword memory performance. However, the Meaning strategy, used alone or in combination with the Position strategy, improved word memory, while the Dual strategy was the most efficient for the pseudoword memory, suggesting that for pseudowords, the Meaning and Position strategies potentiated each other. According to Worthen and Hunt, list-learning mnemonic techniques can be divided into *organization* mnemonic processes, which emphasize intralist associations (i.e., all information to be remembered is linked together), and *elaboration* mnemonic processes, which rely on extralist cues (e.g., meaningfulness, mental imagery) (Worthen and Hunt, 2017). Furthermore, these authors argued that the combined use of the organization and elaboration strategies is the most effective mnemonic. Here, the Position strategy was analogous to an organization mnemonic, while the Meaning strategy was analogous to an elaboration mnemonic. The combination of organization and elaboration mnemonics appeared to be the most effective strategy for pseudoword memory (Figure 3 right). However, this did not apply to word memory, as only the elaboration mnemonic enabled the best performance (Figure 3 left). Moreover, concerning word memory, the Position strategy led to performances as low as those resulting from the use of Listening strategy, which is congruent with the previous findings showing that rote rehearsal is the least effective method for free recall tasks (Herrmann, 1987). Taken together, these two observations strongly suggest that the Meaning strategy was an extremely efficient mnemonic that had the potential to maximize verbal list-learning memory performance.

Finally, we were interested in determining whether the age and sex effects on memory performance could have been mediated by the previous self-reported strategies. We found that aging significantly decreased the likelihood of using any of the advanced strategies, i.e., all strategies other than Listening, for word and pseudoword memory (Table 7), which, in turn, impaired memory performance, as assessed by the CMAs (Table 8). This result strongly suggests that an increase in age produces a nonspecific decrease in the use of advanced strategies. We also observed that being a woman increased the likelihood of using the Meaning and Dual strategies compared to the Position strategy, for word memory (Table 7) and that this effect could to some extent mediate women’s superior word STR performance (Table 8). Thus, the gain in STR performance provided by the words could be specifically related to the fact that women preferentially use the Meaning strategy (alone or in combination with the Position strategy). This result is congruent with previous observations obtained using the CVLT (Kramer et al., 1988, 1997) and supports Andreano and Cahill’s hypothesis that women’s verbal memory advantage depends on encoding at the semantic level (Andreano and Cahill, 2009).

From a neuropsychological perspective, the distinction between normal and pathological aging and the early detection and identification of neurodegenerative diseases are core features of investigations. Since we found that RAVLT scores can capture semantic strategies, we can assume that the RAVLT enables the detection of the early semantic impairment, particularly word-finding difficulties, that characterizes AD (Herlitz and Viitanen, 1991; Croisile et al., 1996; de Lira et al., 2011; Verma and Howard, 2012). Extending this idea, the semantic sensitivity of the RAVLT might explain its previously observed ability to detect the disease in its early stages and, notably, to discriminate AD from MCI (Marra et al., 2000; Estévez-González et al., 2003; Goryawala et al., 2015; Bauer et al., 2018). Moreover, we observed that the word memory advantage of women was mediated by the use of the Meaning strategy (Table 8). This sex-specific mediation can lead to a sex-specific cognitive advantage that compensates for the neurological evolution of AD and, as previously hypothesized by Sundermann et al. (2017), interferes with the early detection of the disease in women. Therefore, when the RAVLT is used for diagnostic purposes, we join the authors in advocating the establishment of sex-specific cutoff scores (Nebel et al., 2018) or sex-adapted performance gaps between healthy individuals and those with dementia (Chapman et al., 2011; Gale et al., 2016).

Since the introduction of the RAVLT in the 1950s, new theories about memory have emerged. One of the most widely recognized is Tulving’s theory, which distinguishes nondeclarative (implicit) memory from declarative (explicit) memory. Moreover, Tulving describes two types of declarative memory, as follows: (1) *episodic memory*, corresponding to personal memories (or contextualized events) and referring to recollection, a key concept related to autonoetic consciousness (a subjective sense of self over time) and (2) *semantic memory*, corresponding to general knowledge and referring to familiarity, which is associated with noetic consciousness (Tulving, 2002). At this stage, it could be useful to relate Tulving’s model of declarative memory to our observations and to discuss the ability of the RAVLT to assess Tulving’s memory systems in light of our results.

Tulving previously specified that “*there is no necessary correlation between behavior and conscious experience and in that sense the traditional research* (i.e., *such sterile situations as list-learning experiments) was not concerned with episodic memory*” (Tulving, 2002). Nevertheless, in the early and mid-1990s, free recall tasks (used as declarative memory tests) and RAVLT scores (DR in particular) began to be associated with the assessment of episodic memory (Roediger, 1990; Litvan et al., 1991; Moffot et al., 1994; Eustache et al., 1995; Kirkby et al., 1995), and this association became increasingly frequent over time, occurring in 13–20% (PubMed) or 33–43% (ScienceDirect) of the RAVLT literature over the last 6 years (keyword cross-search from January 2020). Currently, it is not unusual to find a direct association between the RAVLT and the assessment of verbal episodic memory (Moradi et al., 2017; Barulli et al., 2019; Putcha et al., 2019; Sudo et al., 2019) or even to find the RAVLT being used to validate other verbal episodic memory tests (Morrison et al., 2018). Since episodic memory implies explicit retrieval (Tulving, 1995), the exploration of self-reported strategies is a direct way to investigate whether episodic memory is assessed by the RAVLT. Indeed, a strategy linked to episodic memory could make some autonoetic references to the context of the test (e.g., a participant associating “handle” with the “handle of the door *in the experiment room*”) or an item’s association with a personal contextualized recollection (e.g., a participant associating “fireplace” with the “fireplace *from his childhood*”). This type of autonoetic strategy did not emerge as a regularly used strategy in our results, leading to the conclusion that the RAVLT seems unable to assess episodic memory to a reasonable degree, which is similar to the conclusion reached by previous authors (Tulving, 1985, 2002; Van der Linden, 2004; Desgranges and Eustache, 2011). Consistent with Gavett and Horwitz’s assertion that list-learning test interpretation lacks the construct validity to allowed unbiased estimates of episodic memory ability (Gavett and Horwitz, 2012), our results supplement those reported by Casaletto et al. (2017), who focused on total immediate recall performance during learning trials and concluded that interpreting learning scores as equivalent to episodic memory may be erroneous. To avoid any confusion regarding the purpose of the RAVLT, and considering its widespread use, we encourage the use of simple terminology, i.e., “(supraspan) verbal memory,” to briefly characterize the RAVLT assessment. In addition, it seems appropriate to extend this caution to all supraspan verbal list-learning tests.

Within the framework of Tulving’s memory model, the present results lead to consider the RAVLT scores as being affected by semantic memory. Indeed, the present results show that general knowledge of the meaning of words (the thematic and taxonomic associations used in the Meaning strategy) maximize performance when used as a mnemonic (Figure 3). Moreover, the Meaning strategy was widely used among the participants (Table 5) and was found to mediate women’s advantage regarding the STR of words (Table 8). Therefore, RAVLT performance appears to be highly dependent on this general knowledge and, thus, on semantic memory. Nonetheless, the self-reported strategies explained only 28% (5.6% imputable to the fixed effects) of the variance in RAVLT performance and mediated 2–8% of the age and sex effects on memory performance. These effect sizes suggest that some memory processes escaped the participants’ self-assessment and that the RAVLT scores surely assessed some implicit mechanisms. This hypothesis is compliant with Tulving’s definition of semantic memory, which is declarative knowledge characterized by implicit retrieval (Tulving, 1995). In addition, a quick review of the literature reveals the possible involvement of early implicit encoding processes, such as working memory and executive functions. Although the age and sex effects on memory performance that we observed were adjusted for WMC, other dimensions of working memory and executive functions may be involved. A recent study demonstrated that semantic encoding is automatic in verbal short-term memory (Campoy et al., 2015). Furthermore, the recency effect, which plays a significant role in RAVLT scores, is largely driven by verbal attention (Griffin et al., 2017). The recency effect has been conceptualized in immediate free recall as implicit learning coupled with a particular mode of retrieval that may, but need not, be conscious and explicit (Baddeley and Hitch, 1993). Finally, the present observation that aging nonspecifically decreases the use of advanced self-reported strategies (Table 7) also argues for the involvement of executive functions. This interpretation is consistent with previous observations that normal aging is associated with more difficulty accessing lexico-semantic operations and representations due to a slowdown in executive functions rather than concept loss per se (Baciu et al., 2016). A recent study also showed selective inability to recall RAVLT midlist items in patients with a selective mild executive function deficit (Consonni et al., 2017). Moreover, some authors noted the probable combined effect of attentional and short-term memory processes on the immediate recall score (Gavett and Horwitz, 2012) and a high degree of overlap between verbal memory and executive functioning (Duff et al., 2005). While further studies are needed to clarify the nature of these associations, our results contribute to previous studies suggesting the existence of an association between RAVLT scores and working memory or executive functions.

In conclusion, our results showed that word meaning provides a significant gain in recall performance on the RAVLT. In particular, word meaning enables access to meaning-based memory strategies that optimize verbal memory performance. Moreover, the beneficial role of the Position memory strategy for pseudoword recall was potentiated by a lexical-based memory strategy. These two observations strongly suggest that pseudoword and word-list learning memory performance depends on strategies that are based on general semantic knowledge. Within the framework of Tulving’s declarative memory model, our results indicate that the verbal list-learning test free recall scores are affected by semantic memory. Since the self-reported strategies were not autonoetic, we conclude that the RAVLT is not suitable for episodic memory assessment as it is increasingly referred to in the literature.

## Data Availability Statement

The raw data supporting the conclusions of this article will be made available by the authors, without undue reservation.

## Ethics Statement

The studies involving human participants were reviewed and approved by Comité de Protection des Personnes Nord-Ouest, France. The patients/participants provided their written informed consent to participate in this study.

## Author Contributions

GJ, LZ, and EM contributed to the BIL&GIN database conception. SC contributed to the study design and data organization, performed the statistical analysis, and wrote the first draft of the manuscript. All authors contributed to manuscript redaction and approved the submitted version.

## Conflict of Interest

The authors declare that the research was conducted in the absence of any commercial or financial relationships that could be construed as a potential conflict of interest.

## Footnotes

1 http://www.R-project.org
