# Supplementary material for: Word Meaning Contributes to Free Recall Performance in Supraspan Verbal List-Learning Tests
Source: Front Psychol. 2020 Aug 14;11:2043. doi: 10.3389/fpsyg.2020.02043 (PMC7457129; doi:10.3389/fpsyg.2020.02043)
Supplement: Supplementary file 1 [file Data_Sheet_1.PDF]

Supplementary Material

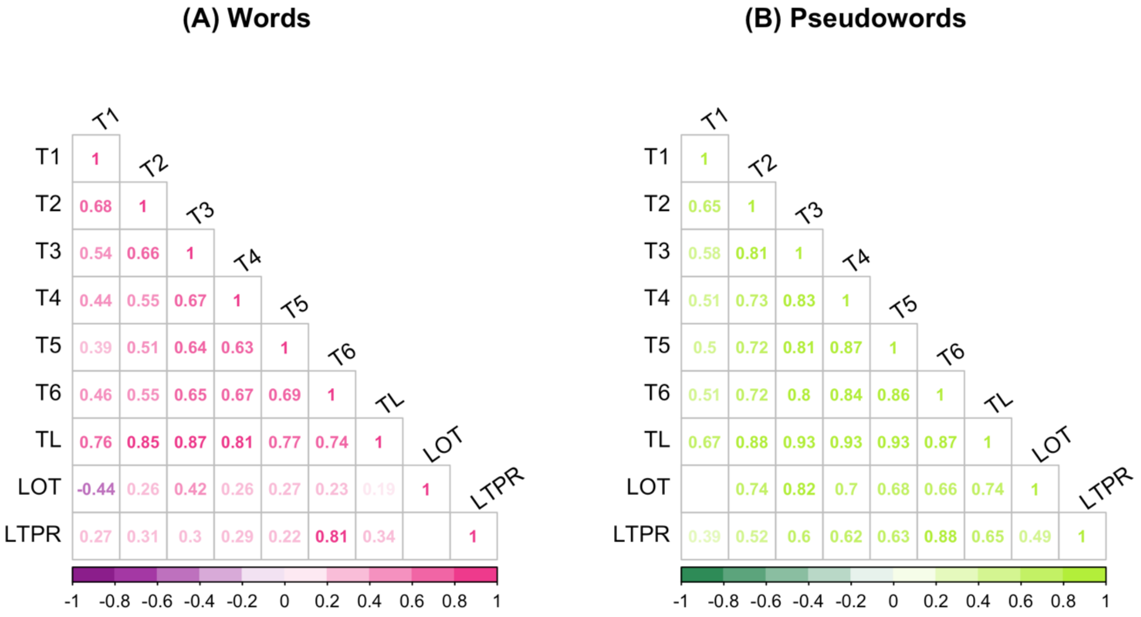

Supplementary figure 1. Pearson's correlation coefficients for word (A) and pseudoword (B) memory scores. Only significant correlations (coefficients whose 95% CI excludes the value of 0) are shown. T=trial; TL=total learning (*i.e.*, the sum of the items recalled from T1 to T5); LOT=learning over trials (*i.e.*, the sum of the items recalled over T2 and T3 minus 2 times the T1); LTPR=long-term percent retention (*i.e.*, the delayed T6 score divided by the maximum score achieved during one of the first 5 learning trials x 100).

All but 2 of the memory scores were highly and significantly correlated. To best evaluate the 3 memory stages separately, we chose the less correlated scores for short-term recall (the recall score at T1) to assess the learning stage and delayed recall. The learning stage can be assessed with the TL or LOT. The delayed recall can be assessed with the T6 or LTPR. The difference between the coefficients of correlation was tested with Williams's test (Revelle, William R. psych: Procedures for Personality and Psychological Research, 2017[1]). T1 had a significantly lower correlation with LOT (words: $r = -0.44$; pseudowords: $r = .09$) than with TL (words: $r = 0.76$; pseudowords: $r = 0.67$), so the LOT was chosen to assess the learning stage (words: $t = 72.49$, $p < 0.0001$; pseudowords: $t = 38.89$, $p < 0.0001$). T1 had a significantly lower correlation with LTPR (words: $r = 0.27$; pseudowords: $r = 0.39$) than with T6 (words: $r = 0.46$; pseudowords: $r = 0.51$), so LTPR was chosen to assess delayed recall (words: $t = 7.45$, $p < 0.0001$; pseudowords: $t = 6.06$, $p < 0.0001$).

---

[1] https://www.scholars.northwestern.edu/en/publications/psych-procedures-for-personality-and-psychological-research
